# Identification of core indicators for the integration of a palliative care approach in hospitals: An international Delphi study

**DOI:** 10.1177/02692163241283540

**Published:** 2024-09-27

**Authors:** Mary Nevin, Sheila Payne, Valerie Smith

**Affiliations:** 1 School of Nursing, Psychotherapy and Community Health, Dublin City University, Dublin, Ireland; 2 International Observatory on End of Life Care, Faculty of Health and Medicine, Lancaster University, Lancaster, UK; 3 School of Nursing and Midwifery, University College Dublin, Belfield, Dublin, Ireland

**Keywords:** Palliative care, Delphi technique, surveys, questionnaire

## Abstract

**Background:**

Healthcare providers working in hospitals have significant exposure to patients with palliative care needs. For many patients, these needs often reflect non-specialist rather than specialist palliative care needs. Embedding a palliative care approach in acute hospital-based care however is challenging.

**Aim:**

To identify core indicators for the integration of a palliative care approach in hospitals.

**Design:**

A Delphi technique used three sequential online survey rounds. Preliminary indicators were identified in a concept analysis of a palliative care approach, and a systematic review of hospital-based healthcare providers’ views of a palliative care approach.

**Participants:**

An international expert panel of three key stakeholder groups (clinicians, researchers, patients/family members) participated in each Delphi round.

**Results:**

The Delphi participants were recruited from 12 countries; 97 individuals responded to round 1, 78 to round 2 and 72 to round 3 (74% overall response rate). Consensus was achieved (defined a priori as >70%) on 32 core indicators of a hospital-based palliative care approach, with five structural indicators (relating to infrastructure and governance), 21 organisational indicators (relating to clinical care processes) and six staff indicators (relating to training and support for healthcare providers).

**Conclusions:**

This study offers multi-level guidance for clinical practice, policy and research related to integration of a palliative care approach in hospitals, based on evidence and international consensus from major stakeholder groups. These core indicators provide a means to assess, review and communicate the core elements of a palliative care approach in hospitals.

**What is already known about this topic?**

- It is recommended that healthcare providers should have at a minimum, a basic knowledge of palliative care.
- Healthcare providers working in hospitals care have significant exposure to patients with palliative care needs but meeting these needs remains a challenge.
- The Delphi technique is a well-established method of eliciting the views of stakeholders and obtaining consensus.

**What this paper adds?**

- This paper describes the identification of core indicators for the integration of a palliative care approach in hospitals using a Delphi technique.
- Thirty-two core indicators were identified comprising 5 core structural indicators (relating to infrastructure and governance), 21 core organisational indicators (relating to clinical care processes) and 6 core staff indicators (relating to training and support for healthcare providers).

**Implications for practice, theory or policy?**

- These core indicators can be used as a basis to support multi-level integration of a palliative care approach within acute hospital-based care.
- Further research is needed on optimal approaches to implementing a palliative care approach in hospitals

## Background

It is well established that the rapidly rising demand for palliative care globally will require optimal utilisation of all components of service provision within health systems.^1,2^ Healthcare providers working in acute hospital-based healthcare provision have an important role in the care of people with palliative care needs.^3^ Estimates indicate that the likelihood of dying in hospital varies between countries but is generally high.^4^ Acute hospital-based care however has occupied a relatively ambiguous position within the provision of palliative and end of life care,^5,6^ and there are mixed views whether this care environment is a preferential place to die.^7^ Palliative care is an approach that improves the quality of life of people and families facing the problems associated with life-limiting illness. The palliative care approach focuses on the prevention and relief of suffering by means of assessing and treating pain and other physical, psychosocial or spiritual problems and all healthcare providers should have, at a minimum, basic knowledge of palliative care.^8,9^ Although competencies are available to support health care staff to self-assess, develop and maintain skills, knowledge and attributes required for the provision of palliative care,^10^ the reporting of unmet palliative care needs in hospitals continues to be a concern where the momentum of care is towards rescue, resuscitation and lifesaving interventions.^11–14^ Evidence, however, suggests that interventions which reflect a palliative care approach cannot be easily packaged and implemented within acute hospital-based care without careful attention being given to the complexities of the culture and context of that environment.^15^ Studies of healthcare providers in hospitals working outside of specialist palliative care services have highlighted difficulties such as recognising those with palliative care needs and engaging in conversations with patients regarding poor prognosis,^16–18^ complex and uncertain disease trajectories,^12^ role uncertainty and integrating palliative care within an acute care workload,^8,9^ as issues which contribute to difficulties in providing palliative care in this setting. Increasing the capacity to support a palliative care approach for those with chronic and life limiting conditions, within acute hospital-based clinical practice necessitates a major change in core assumptions about the organisation and provision of acute care, requiring a multi-level approach to change.^12,19^

The term ‘integrated palliative care’ is described as bringing together aspects such as organisational, clinical and service elements to provide continuity of care between all those involved in the care network of patients receiving palliative care.^20^ The body of evidence pointing to the integration of palliative care early within acute care largely emanates from intervention studies relating to the integration of specialist palliative care services.^21–23^ Furthermore, although there have been calls to measure and evaluate the effectiveness of a palliative care approach,^24–26^ in principle it is very difficult to evaluate quality of care without clear understanding as to what that care consists of in practice. Clinical indicators are a means to achieve this and are used for a variety of purposes to support the quality and safety of health services.^27^ For the purposes of this study, an ‘indicator’ of a palliative care approach is considered a statement that describes an aspect of a palliative care approach which is relevant within the hospital care environment. The processes for developing previous indicators relating to palliative care were examined to identify areas for improvement and guide the development of indicators of a palliative care approach in hospitals in the current study. In a previously conducted review of palliative care quality indicators,^28^ only one example of specialist palliative care hospital-based indicators was identified.^29^ Most of the quality indicators of palliative care identified in this review reflected care processes and outcomes, with very few that reflected structural aspects which support quality palliative care provision.^28^ Therefore, important contextual concerns such as infrastructure and resources which will affect the integration of palliative care were not considered.^30^ Furthermore, it has long been established that the perspectives of patients and families should be encouraged in the definition and adoption of indicators of good palliative care from all dimensions of care.^31,32^ However there was little evidence of patient and/or family involvement in the development of previous hospital-based palliative care quality indicators,^29^ and little attention has been given to the patient and family perspective in developing guidance for the integration of a palliative care approach in hospitals.^33^ The aim of this study was to achieve consensus on essential (core) indicators which provides direction for hospital provision to integrate a palliative care approach within all levels of the organisation from the perspectives of all key stakeholders.

## Methods

To achieve the study’s aim, a three-round international online Delphi survey was used. This is a well-established formal consensus method, which enabled the inclusion of international experts in a cost effective and efficient manner.^34^ The Delphi method is especially useful where participants can express views anonymously in an iterative process, without being unduly influenced by Hasson and Keeney^35^ and Vernon^36^ In doing so the perspectives of multiple key stakeholders associated with a palliative care approach in hospitals could be ascertained efficiently, and over a relatively short duration of time. This study was guided by Conducting and REporting Delphi Studies (CREDES).^37^

### Identification of initial indicators

**Figure 1.**
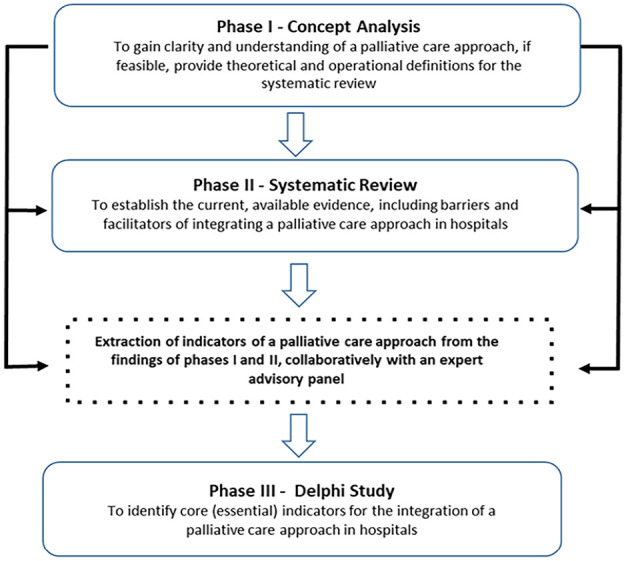
Research phases to identify core indicators for the integration of a palliative care approach in hospitals.

This study had three distinct, yet complimentary phases (see Figure 1). Phase I involved a principle-based concept analysis of a palliative care approach. This analysis of 124 sources of evidence yielded information about terminology associated with a palliative care approach, how this approach is currently understood and described the key attributes of a palliative care approach under the four broad philosophical perspectives of epistemology, pragmatism, logistics and linguistics.^9^ Phase II involved a qualitative systematic review of 37 studies,^8^ representing the views of 985 healthcare providers in hospitals which reported on the experiences, barriers and facilitators of integrating a palliative care approach to understand relevant contextual issues.^8^

Phase III began by scrutinising the findings from phases I and II, separately, rewording the findings into short summary statements which relate to indicators of a palliative care approach in the hospital setting, and deriving clear, concise indicator statements to develop the initial indicators for use in a Delphi survey. After deriving the initial indicator statements, these were further scrutinised for similarity and overlap and combined, as appropriate to provide the complete list of preliminary indicators of a palliative care approach for hospitals. The indicators were then divided into three sections. Those indicators that related to the infrastructural or strategic governance systems within a hospital were combined into one category. Indicators related to clinical policies, guidelines or care processes were combined into a second category. Finally, any indicators that related to activities of individual hospital staff were categorised together.

To enhance rigor, an advisory panel was established to provide input and advice on the initial indicators and the round one Delphi survey instrument. The panel consisted of a hospital-based clinician, a palliative care researcher, a person with a chronic illness, a Delphi researcher, the medical director of a clinical audit department in a large acute care hospital setting, and an end-of-life care co-ordinator of a large acute care hospital setting. The role of the panel was to review the list of preliminary indicators and to provide feedback in accordance with their individual expertise in relation to their structure, clarity, appropriateness and readability. The development (extraction, scrutiny, categorisation, advisory and refinement) process culminated in the **r**ound 1 survey. The **r**ound 1 survey included 34 indicators related to a palliative care approach in the hospital setting which were subdivided into 5 structural, 18 organisational and 11 staff indicators. Structural indicators referred to the physical and organisational structures and facilities, and the strategic governance systems within the hospital. Organisational indicators referred to clinical policies, guidelines and clinical care processes, while staff indicators referred to the care providers in the hospital setting.

### Population, sample and recruitment

For the purposes of this study, the Delphi technique was used to elicit broad expertise and perspectives on the topic. It is advantageous to include heterogenous groups of experts with a wide understanding of the area in question.^36^ Therefore, three stakeholder groups of experts were identified as; service users of a palliative care approach in hospitals, hospital-based healthcare providers and researchers/policy makers in palliative care. The Delphi survey was designed online and made available using SurveyMonkey©. Round 3 recruitment was completed in June 2019. Invitation emails, inclusive of a link to the online Delphi survey, were sent to the ‘contact us’ details of palliative care advocacy organisations, and chronic illness organisations internationally with a request to disseminate the invitation email to their members. Furthermore, the contact details of the first authors of all publications sourced for phases I and II, and any policy documents relating to a palliative care approach were identified, and an invitation email was distributed to these individuals. Snowball sampling was achieved by asking participants to forward the link to others who might have the necessary expertise to participate in the study. Informed consent was provided by participants by ticking the *‘I consent to take part’* box which was made available in each Delphi round. Ethical approval to conduct the study was granted by the Research Ethics Committee of the lead author’s University.

### Data collection

For rounds 1 and 2, a 5-point categorical rating scale ranging from *Strongly Agree* to *Strongly Disagree* was utilised to ascertain participants’ level of agreement as to whether each indicator statement presented was considered essential (core) for a palliative care approach to occur in hospital. Consensus in rounds 1 and 2 was based on ⩾70% of the participants in at least two of the three stakeholder groups indicating either *strongly agree* or *agree* to each indicator statement. This was to ensure that one stakeholder group did not dominate the consensus.^38^ This definition of consensus (⩾70%) is also consistent with previously conducted Delphi studies in palliative care.^39,40^ Participants were also offered the opportunity, in round 1 to propose additional core indicators not already in the list. Any newly suggested indicators were added to round 2. To determine ‘new indicators’ suggested in round 1, the suggestions were read and re-read to identify overlap and potential duplication of content and words, and terms that conveyed similar meaning were grouped.^41^ Respondents to round 1 were sent the round 2 survey and were invited to re-rate all indicators provided to them in round 1, and rate any newly added indicators using the same round 1 rating scale. The opportunity to revise previous scores in light of previous ratings is an important element in consensus in a Delphi study.^42,43^ Therefore, in round 2 participants were presented with a table alongside each indicator with each group’s round 1 percentage level of agreement. Aligning with consensus methods, only those indicators that reached at least 70% agreement, in at least two participant groups at the end of round 2, were sent forward to round 3. To further channel participants into deciding whether an indicator was considered core or not, in round 3, the 5-point Likert scale was replaced by YES/NO response options whereby YES is chosen if a participant deems this absolutely essential as a core indicator for the integration of a palliative care approach in acute hospitals. Consensus on the final core indicators was ⩾70% agreement ‘YES’ in at least two participant groups.

### Data analysis

To maintain momentum in the process, and guard against participant apathy, the results for each round were analysed and the links for access to the rounds 2 and 3 surveys were circulated to those participants who participated in the previous round one week after the official close of that round. Two reminder emails were sent to non-responders during rounds 2 and 3 1 week apart. SPSS^®^ version 25 was used to analyse the data in each round which included the response rates for each round (counts), percentages for each level of agreement, at group level, for each indicator (i.e. strongly disagree to strongly agree), and median scores, which are recommended to be reported in Delphi studies.^44^

## Results

### Participation in the study

Ninety-seven individuals from 12 countries responded to round 1. Of these, 78 (82%) responded to round 2, and of these 72 responded in round 3 (overall response rate of 74%). The characteristics of the respondents in round 1 are presented in Table 1. The breakdown of each expert group over the three rounds is provided in Table 2, while Table 3 provides details of the countries of origin in each round of this Delphi study.

**Table 1.** Characteristics of round 1 Delphi participant groups.

Stakeholder group	Participants		*N*	%
1. Patients and families *N* = 25	Adult who has a chronic or serious illness with experience of care in a hospital		10	10.3
	Family/Carer of an adult who has a chronic or serious illness		8	8.2
	Representative or member of an advocacy group for adults with a chronic or serious illness		7	7.2
2. Hospital-based healthcare providers *N* = 52 *(of the 45 clinicians;* *15 = Specialist palliative care (33%)* *28 = a palliative care approach (62%; 2 didn’t answer)*	Consultant hospital Doctor	Palliative Medicine 6 Respiratory Medicine 3 Geriatric 2 Anaesthesia and Pain 1 Internal Medicine 1	13	13.4
	Hospital-based Nurse	Ward level Nurse 5 Clinical Nurse Manager 1 Nurse Specialist 15 *(not specified 4, respiratory 2, emergency 2, palliative care 5, ‘consultant’ 2)*	21	21.6
	Other hospital healthcare worker	Not Indicated 2 Medical social worker 1 Physiotherapist (2 cystic fibrosis) 7 Physiologist 1	11	11.3
	Hospital management	Director Nursing 1 Assistant Director of Nursing 2 End Of Life Co-ordinator 2 Coordinator Integrated care unit 1 Manager Palliative Care 1	7	7.2
3. Researchers or Policy makers in palliative care *N* = 20	Researcher/Academic in palliative care		20	20.6
Total responses			97	100

**Table 2.** Summary of Delphi group participation.

Delphi rounds	Total number of participants per round	Stakeholder groups					
		Those who have experience of a palliative care approach		Hospital-based healthcare providers		Researchers or policy makers in palliative care	
	*N*	*N*	%	*N*	%	*N*	%
Round 1	97	25	25.8	52	53.6	20	20.6
Round 2	78	21	26.9	39	50.0	18	23.1
Round 3	72	21	29.2	33	45.8	18	25.0

**Table 3.** Country of origin per Delphi round.

Results	Round 1		Round 2		Round 3	
Country of origin	*N*	%	*N*	%	*N*	%
Canada	3	3.1	2	2.6	2	2.8
Denmark	1	1.0	1	1.3	1	1.4
Finland	1	1.0	1	1.3	1	1.4
Ireland	59	60.8	47	60.3	43	59.7
Italy	1	1.0	1	1.3	1	1.4
New Zealand	11	11.3	9	11.5	8	11.1
Norway	1	1.0	1	1.3	1	1.4
Portugal	2	2.1	1	1.3	1	1.4
Spain	1	1.0	1	1.3	1	1.4
Sweden	9	9.3	6	7.7	5	6.9
United Kingdom	7	7.2	7	9.0	7	9.7
United States of America	1	1.0	1	1.3	1	1.4
Total	97	100.0	78	100.0	72	100.0
No. of Countries per round	12		12		12	

### Delphi rounds

#### Round 1 Delphi results

Table 4 presents the results of the 34 indicators rated in round 1. Indicators that achieved ⩾70% agreement (either *strongly agree* or *agree*) in at least two participant groups were deemed to have achieved consensus. Consensus was achieved for 20 of the 34 round 1 indicators, specifically 1 of the 5 structural indicators, 13 of the 18 organisational indicators and 6 of the 11 staff indicators.

**Table 4.** Delphi round 1 results.

Indicator		Patient/carer/advocate *N* = 25	Hospitalist *N* = 52	Researcher *N* = 20
Structural indicators		Percentage level of agreement (⩾70% strongly agree or agree)		
1	Palliative care provision at all levels is clearly defined and communicated within the strategic framework/service plan of the hospital	76.0	63.0	55.0
2	Evaluation of hospital service provision includes quality measures related to a palliative approach	80.0	58.7	61.1
3	Patient and family reported experiences of a palliative approach are included in service provision evaluation	76.0	59.1	72.2
4	The strategic palliative care service plan of the hospital details the resources, funding, staff training and support necessary to provide an effective palliative approach for patients with life limiting illness	68.0	54.6	66.6
5	A commitment to ensuring that adequate infrastructural resources (i.e. the basic physical and structural facilities) that support patient and family privacy are included in the strategic goal to integrate a palliative approach within a hospital	75.0	65.9	55.6
	Organisational indicators			
6	Organisational and care practices are structured in a way that supports a palliative approach to care provision in combination with acute care	65.2	81.0	77.7
7	Hospital policies and guidelines associated with a palliative approach emphasise early and timely assessment of palliative care needs	73.9	81.0	66.6
8	Guidelines relating to a palliative approach are incorporated into clinical disease management frameworks and protocols relating to life limiting illnesses	65.2	67.5	83.3
9	Guidelines relating to a palliative approach are adapted according to the type of illness/patient within clinical disease management frameworks and protocols relating to life limiting illnesses	73.9	52.8	61.1
10	Non-specialist palliative care provision is structured to reflect the knowledge levels of those with greater experience and/or exposure to patients with life limiting illness	63.7	64.8	44.4
11	Integrating a palliative approach requires care providers routinely assess the palliative care needs of patients with life limiting illness	81.8	77.8	55.6
12	Integrating a palliative approach requires care providers to manage palliative pain and other symptoms experienced by patients with life limiting illness	72.7	86.4	77.7
13	Integrating a palliative approach requires that patients with life limiting illness and their families receive timely and sensitive communication	77.3	83.7	72.2
14	Integrating a palliative approach requires care providers requires that patients with life limiting illness receive timely information about their illness trajectory (i.e. likely course of the illness) and prognosis (i.e. likely outcome of the illness)	72.8	75.0	61.1
15	Integrating a palliative approach requires that patients with life limiting illness receive psychosocial support (the influence that attitudes/behaviours and the surrounding social environment have on patients physical and mental wellness and ability to function)	72.7	70.2	77.8
16	Integrating a palliative approach requires that patients with life limiting illness receive spiritual/existential support (relating to life meaning, purpose or value)	68.2	64.8	66.6
17	Integrating a palliative approach requires that patients with life limiting illness are facilitated and supported to engage in advance care planning discussions	59.1	78.3	55.5
18	Integrating a palliative approach requires that patients with life limiting illness receive compassionate end of life care	81.8	91.6	88.9
19	Integrating a palliative approach requires that formal mechanisms are in place in clinical departments/wards to facilitate shared decision making between doctors and nurses caring for patients/families with life limiting illness	77.3	77.1	61.1
20	Integrating a palliative approach requires that formal mechanisms are in place in clinical departments/wards to ensure goals of care for patients with life limiting illness are clearly communicated between all staff caring for the patient and their family	81.8	72.9	61.1
21	The hospital has a multidisciplinary specialist palliative care service in place	81.8	81.2	83.3
22	A structured and standardised care pathway exists between clinical staff and specialist palliative care, to support mutual decision making and organisation of care processes, including clarification of roles and responsibilities of care	76.1	72.9	50.0
23	Specialist palliative care services have a clearly identified role in the continued training and support of other care staff	72.7	73.0	83.3
	Staff indicators			
24	Staff caring for patients with life limiting illness receive evidence-based training on palliative care principles, assessment and care management	83.4	66.7	76.5
25	Early career staff, and newly recruited staff caring for patients with life limiting illness receive evidence-based training on palliative care principles as part of induction training	83.3	58.4	70.6
26	Staff receive training on recognising and being able to assess the palliative care needs of patients	88.2	69.5	82.3
27	Staff receive training on a palliative approach to patients’ pain and symptom control	82.3	72.2	88.3
28	Staff receive communication training regarding the needs of patients and their families with a life limiting illness	88.2	72.2	70.6
29	Staff receive training on advance care planning	82.4	61.1	64.7
30	Staff receive training on compassionate end of life care	88.2	74.2	82.4
31	The content of training is organised to meet the knowledge requirements of staff with greater experience of or exposure to patients with life limiting illness	70.6	50.0	58.8
32	Formal mechanisms are in place in clinical departments/wards to facilitate multi-disciplinary learning between all relevant staff caring for patients with life limiting illness	76.4	61.1	52.9
33	Formal peer support (such as case review meetings) is used to support staff caring for patients with life limiting illness	82.3	55.6	52.9
34	Informal peer support (such as corridor conversations) is used to support staff caring for patients with life limiting illness	58.8	69.4	64.7

#### Round 2 Delphi results

Participants were provided with the results of round 1 for review and re-rating. From the ‘additional indicators’ section of round 1, 12 new indicators were identified and aligned to the relevant indicator category. This produced one structural, nine organisational and two new staff category indicators, providing, overall, a list of 46 indicators for rating in round 2 (6 structural, 27 organisational and 13 staff indicators). In round 2, 41 of the 46 indicators achieved consensus and were thus retained for re-rating in round 3. The five indicators that did not achieve consensus were excluded from round 3. Table 5 provides the results of round 2. Indicators in *italics* are the 12 new indicators identified from round 1.

**Table 5.** Delphi round 2 results -level of agreement and decision for inclusion in final round.

Indicator (those in italics are new indicators added following round 1)*		Patient/carer/advocate *N* = 21	Hospitalist *N* = 39	Researcher *N* = 18	Decision after round 2
Structural Indicators		Percentage Level of Agreement (⩾70% strongly agree or agree in at least two stakeholder groups)			
1	Palliative care provision at all levels is clearly defined and communicated within the strategic framework/service plan of the hospital	71.4	84.6	88.9	Retained
2	Evaluation of hospital service provision includes quality measures related to a palliative approach	90.5	59.0	100.0	Retained
3	Patient and family reported experiences of a palliative approach are included in service provision evaluation	95.2	66.7	83.3	Retained
4	Complaints relating to care for patients and families with life limiting illness are reviewed regularly by hospital management*	85.9	84.6	83.4	Retained
5	The strategic palliative care service plan of the hospital details the resources, funding, staff training and support necessary to provide an effective palliative approach for patients with life limiting illness	61.9	53.8	77.7	Excluded
6	A commitment to ensuring that adequate infrastructural resources (i.e. the basic physical and structural facilities) that support patient and family privacy are included in the strategic goal to integrate a palliative approach within a hospital	90.4	84.2	83.3	Retained
	Organisational indicators				
7	Organisational and care practices are structured in a way that supports a palliative approach to care provision in combination with acute care	80.0	76.3	94.4	Retained
8	Clear systems and processes for co-ordination of care, dealing with uncertainty in acute illness in those with poor prognosis, future care planning (advance care planning and anticipatory clinical management planning) and recognition and management of dying are in place*	90.0	73.7	88.9	Retained
9	Hospital policies and guidelines associated with a palliative approach emphasise early and timely assessment of palliative care needs	80.0	82.1	83.3	Retained
10	When appropriate referral to the Specialist Palliative Care multidisciplinary team occurs in a timely manner*	90.0	86.9	88.9	Retained
11	Guidelines relating to a palliative approach are incorporated into clinical disease management frameworks and protocols relating to life limiting illnesses	75.0	77.0	94.5	Retained
12	Guidelines relating to a palliative approach are adapted according to the type of illness/patient within clinical disease management frameworks and protocols relating to life limiting illnesses	75.0	63.2	83.3	Retained
13	Non-specialist palliative care provision is structured to reflect the knowledge levels of those with greater experience and/or exposure to patients with life limiting illness	70.0	56.4	55.6	Excluded
14	Integrating a palliative approach requires care providers routinely assess the palliative care needs of patients with life limiting illness	80.0	87.2	77.8	Retained
15	Integrating a palliative approach requires care providers to manage palliative pain and other symptoms experienced by patients with life limiting illness	80.0	94.9	83.3	Retained
16	Integrating a palliative approach requires that patients with life limiting illness and their families receive timely and sensitive communication	85.0	82.0	88.9	Retained
17	Integrating a palliative approach requires care providers requires that patients with life limiting illness receive timely information about their illness trajectory (i.e. likely course of the illness) and prognosis (i.e. likely outcome of the illness)	75.0	64.1	72.2	Retained
18	Integrating a palliative approach requires that patients with life limiting illness receive psychosocial support (the influence that attitudes/behaviours and the surrounding social environment have on patients physical and mental wellness and ability to function)	80.0	81.8	83.3	Retained
19	Integrating a palliative approach requires that patients with life limiting illness receive spiritual/existential support (relating to life meaning, purpose or value)	80.0	63.2	77.7	Retained
20	Integrating a palliative approach requires that patients with life limiting illness are facilitated and supported to engage in advance care planning discussions	75.0	71.8	66.7	Retained
21	Appropriate and dedicated space/room is provided in non-specialist palliative care settings for patients and families to spend time together, and where private conversations can occur regarding care needs*	85.0	71.8	88.9	Retained
22	Integrating a palliative approach requires that patients with life limiting illness receive compassionate end of life care	95.0	97.3	94.5	Retained
23	Non-specialist palliative care providers update patients’ advance care preferences on a regular basis and post life threatening exacerbation*	75.0	59	77.8	Retained
24	Clear systems and processes for rapid end of life transfers to preferred place of care for those who are dying*	90.0	66.7	77.7	Retained
25	Non-specialist staff are aware of palliative community services when discharging a patient*	65.0	86.8	83.4	Retained
26	People with life-limiting conditions using hospital services have prioritised readmission to services if required after discharge*	80.0	52.3	55.6	Excluded
27	The family are considered the unit of care, both informal and formal supports are available to the family and when this involves children age-appropriate information and support be available*	75.0	82.1	100	Retained
28	Integrating a palliative approach requires that formal mechanisms are in place in clinical departments/wards to facilitate shared decision making between doctors and nurses caring for patients/families with life limiting illness	80.0	82.0	77.8	Retained
29	Integrating a palliative approach requires that formal mechanisms are in place in clinical departments/wards to ensure goals of care for patients with life limiting illness are clearly communicated between all staff caring for the patient and their family	90.0	87.1	72.2	Retained
30	The hospital has a multidisciplinary specialist palliative care service in place	85.0	97.5	94.1	Retained
31	A structured and standardised care pathway exists between clinical staff and specialist palliative care, to support mutual decision making and organisation of care processes, including clarification of roles and responsibilities of care	80.0	82.0	66.6	Retained
32	The hospital has a multidisciplinary specialist palliative care consultation team in place, that is not only a ‘service’ but a service that includes consultations to non-palliative care specialists*	75.0	73.7	88.9	Retained
33	Specialist palliative care services have a clearly identified role in the continued training and support of other care staff	85.0	84.6	83.3	Retained
	Staff indicators				
34	Staff caring for patients with life limiting illness receive evidence-based training on palliative care principles, assessment and care management	85.0	83.7	88.9	Retained
35	Specialist and non-specialist palliative care providers collaboratively develop and facilitate disease specific education within a speciality*	80.0	62.1	72.2	Retained
36	Early career staff, and newly recruited staff caring for patients with life limiting illness receive evidence-based training on palliative care principles as part of induction training	70.0	67.5	88.9	Retained
37	Staff receive training on recognising and being able to assess the palliative care needs of patients	80.0	78.3	94.5	Retained
38	Staff receive training on a palliative approach to patients’ pain and symptom control	90.0	86.4	88.9	Retained
39	Staff receive communication training regarding the needs of patients and their families with a life limiting illness	90.0	83.8	100.0	Retained
40	Staff receive training on advance care planning	85.0	62.1	88.8	Retained
41	Staff receive training on compassionate end of life care	95.0	86.1	94.5	Retained
42	The content of training is organised to meet the knowledge requirements of staff with greater experience of or exposure to patients with life limiting illness	60.0	64.8	61.1	Excluded
43	Formal mechanisms are in place in clinical departments/wards to facilitate multi-disciplinary learning between all relevant staff caring for patients with life limiting illness	80.0	70.3	66.7	Retained
44	Case presentations at hospital ‘grand rounds’ include difficult palliative care cases*	50.0	76.3	77.8	Retained
45	Formal peer support (such as case review meetings) is used to support staff caring for patients with life limiting illness	80.0	71.1	66.6	Retained
46	Informal peer support (such as corridor conversations) is used to support staff caring for patients with life limiting illness	45.0	81.6	61.1	Excluded

#### Round 3 Delphi results

Table 6 provides the results of round 3. In round 3, the 41 indicators retained from round 2 were re-rated whereby participants were asked to indicate either ‘yes’ or ‘no’ if the indicator should be included in the final core set. Consensus (⩾70% agreement ‘yes’ in at least two participant groups) was achieved for 32 of these reflecting the final core indicators for the integration of a palliative care approach in acute hospitals (5 structural, 21 organisational and 6 staff indicators; Table 7). All 32 core indicators achieved consensus in the patient/carer/advocate stakeholder group. While 26 of the 32 core indicators achieved consensus in all three stakeholder groups. Furthermore, of the 34 indicators that were presented for rating in round 1, 21 were retained following the final round indicating a high degree of stability between Delphi rounds.

**Table 6.** Delphi round 3 results -level of agreement.

Indicator		Patient/carer/advocate *N* = 21	Hospitalist *N* = 33	Researcher *N* = 18	Decision after round 3
Structural indicators		Percentage level of agreement (⩾70% who answered YES in at least two stakeholder groups)			
1	Palliative care provision at all levels is clearly defined and communicated within the strategic framework/service plan of the hospital	71.4	87.9	94.1	Retained
2	Evaluation of hospital service provision includes quality measures related to a palliative approach	100.0	69.7	83.3	Retained
3	Patient and family reported experiences of a palliative approach are included in service provision evaluation	100.0	66.7	83.3	Retained
4	Complaints relating to care for patients and families with life limiting illness are reviewed regularly by hospital management	90.5	93.9	77.8	Retained
5	A commitment to ensuring that adequate infrastructural resources (i.e. the basic physical and structural facilities) that support patient and family privacy are included in the strategic goal to integrate a palliative approach within a hospital	85.7	84.8	83.3	Retained
	Organisational indicators				
6	Organisational and care practices are structured in a way that supports a palliative approach to care provision in combination with acute care provision	90.5	81.8	88.2	Retained
7	Clear systems and processes for co-ordination of care, dealing with uncertainty in acute illness in those with poor prognosis, future care planning (advance care planning and anticipatory clinical management planning) and recognition and management of dying are in place	100.0	87.9	94.1	Retained
8	Hospital policies and guidelines associated with a palliative approach emphasise early and timely assessment of palliative care needs	76.2	81.8	94.4	Retained
9	When appropriate referral to the Specialist Palliative Care multidisciplinary team occurs in a timely manner	85.7	93.9	100.0	Retained
10	Guidelines relating to a palliative approach are incorporated into clinical disease management frameworks and protocols relating to life limiting illnesses	70.0	84.8	94.1	Retained
11	Guidelines relating to a palliative approach are adapted according to the type of illness/patient within clinical disease management frameworks and protocols relating to life limiting illnesses	70.0	42.4	58.8	Excluded
12	Integrating a palliative approach requires care providers routinely assess the palliative care needs of patients with life limiting illness	90.5	81.3	83.3	Retained
13	Integrating a palliative approach requires care providers to manage palliative pain and other symptoms experienced by patients with life limiting illness	85.7	97.0	94.4	Retained
14	Integrating a palliative approach requires that patients with life limiting illness and their families receive timely and sensitive communication	95.2	100.0	100.0	Retained
15	Integrating a palliative approach requires care providers requires that patients with life limiting illness receive timely information about their illness trajectory (i.e. likely course of the illness) and prognosis (i.e. likely outcome of the illness)	75.0	54.5	52.9	Excluded
16	Integrating a palliative approach requires that patients with life limiting illness receive psychosocial support (the influence that attitudes/behaviours and the surrounding social environment have on patients physical and mental wellness and ability to function)	76.2	81.8	94.4	Retained
17	Integrating a palliative approach requires that patients with life limiting illness receive spiritual/existential support (relating to life meaning, purpose or value)	52.4	54.5	52.9	Excluded
18	Integrating a palliative approach requires that patients with life limiting illness are facilitated and supported to engage in advance care planning discussions	66.7	69.7	62.5	Excluded
19	Appropriate and dedicated space/room is provided in non-specialist palliative care settings for patients and families to spend time together, and where private conversations can occur regarding care needs	90.5	78.8	77.8	Retained
20	Integrating a palliative approach requires that patients with life limiting illness receive compassionate end of life care	85.7	97.0	100.0	Retained
21	Non-specialist palliative care providers update patients’ advance care preferences on a regular basis and post life threatening exacerbation	70.0	45.5	70.6	Retained
22	Clear systems and processes for rapid end of life transfers to preferred place of care for those who are dying	100.0	72.7	76.5	Retained
23	Non-specialist staff are aware of palliative community services when discharging a patient	90.5	81.8	88.2	Retained
24	The family are considered the unit of care, both informal and formal supports are available to the family and when this involves children age-appropriate information and support be available	76.2	81.8	88.2	Retained
25	Integrating a palliative approach requires that formal mechanisms are in place in clinical departments/wards to facilitate shared decision making between doctors and nurses caring for patients/families with life limiting illness	90.5	75.8	76.5	Retained
26	Integrating a palliative approach requires that formal mechanisms are in place in clinical departments/wards to ensure goals of care for patients with life limiting illness are clearly communicated between all staff caring for the patient and their family	90.5	78.8	94.1	Retained
27	The hospital has a multidisciplinary specialist palliative care service in place	81.0	93.9	88.9	Retained
28	A structured and standardised care pathway exists between clinical staff and specialist palliative care, to support mutual decision making and organisation of care processes, including clarification of roles and responsibilities of care	90.0	81.8	52.9	Retained
29	The hospital has a multidisciplinary specialist palliative care consultation team in place, that is not only a ‘service’ but a service that includes consultations to non-palliative care specialists	85.7	62.5	82.4	Retained
30	Specialist palliative care services have a clearly identified role in the continued training and support of other care staff	76.2	90.9	88.9	Retained
	Staff indicators				
31	Staff caring for patients with life limiting illness receive evidence-based training on palliative care principles, assessment and care management	85.7	90.9	100.0	Retained
32	Specialist and non-specialist palliative care providers collaboratively develop and facilitate disease specific education within a speciality	66.7	42.4	52.9	Excluded
33	Early career staff, and newly recruited staff caring for patients with life limiting illness receive evidence-based training on palliative care principles as part of induction training	81.0	66.7	88.2	Retained
34	Staff receive training on recognising and being able to assess the palliative care needs of patients	90.5	87.9	100.0	Retained
35	Staff receive training on a palliative approach to patients’ pain and symptom control	90.5	90.9	94.4	Retained
36	Staff receive communication training regarding the needs of patients and their families with a life limiting illness	85.7	87.9	100.0	Retained
37	Staff receive training on advance care planning	61.9	54.5	76.5	Excluded
38	Staff receive training on compassionate end of life care	85.7	97.0	100.0	Retained
39	Formal mechanisms are in place in clinical departments/wards to facilitate multi-disciplinary learning between all relevant staff caring for patients with life limiting illness	66.7	42.4	35.3	Excluded
40	Case presentations at hospital ‘grand rounds’ include difficult palliative care cases	66.7	51.5	55.6	Excluded
41	Formal peer support (such as case review meetings) is used to support staff caring for patients with life limiting illness	76.2	45.5	50.0	Excluded

**Table 7.** Core indicators for the integration of a palliative care approach in hospitals.

Structural core indicators	
1	Palliative care provision at all levels is clearly defined and communicated within the strategic framework/service plan of the hospital
2	Evaluation of hospital service provision includes quality measures related to a palliative care approach
3	Patient and family reported experiences of a palliative care approach are included in service provision evaluation
4	Complaints relating to care for patients and families with life limiting illness are reviewed regularly by hospital management
5	A commitment to ensuring that adequate infrastructural resources (i.e. the basic physical and structural facilities) that support patient and family privacy are included in the strategic goal to integrate a palliative care approach within a hospital
	Organisational core indicators
6	Organisational and care practices are structured in a way that recognises the need, and supports a palliative care approach in combination with acute care
7	Clear systems and processes for co-ordination of care, dealing with uncertainty in acute illness in those with poor prognosis, future care planning (advance care planning and anticipatory clinical management planning) and recognition and management of dying are in place
8	Hospital policies and guidelines associated with a palliative care approach emphasise early and timely assessment of palliative care needs
9	When appropriate referral to the specialist palliative care multidisciplinary team occurs in a timely manner
10	Guidelines relating to a palliative care approach are incorporated into clinical disease management frameworks and protocols relating to life limiting illnesses
11	Integrating a palliative care approach requires care providers to routinely assess the palliative care needs of patients with life limiting illness
12	Integrating a palliative care approach requires care providers to manage palliative pain and other symptoms experienced by patients with life limiting illness
13	Integrating a palliative care approach requires that patients with life limiting illness and their families receive timely and sensitive communication
14	Integrating a palliative care approach requires that patients with life limiting illness receive psychosocial support (the influence that attitudes/behaviours and the surrounding social environment have on patients physical and mental wellness and ability to function)
15	Appropriate and dedicated space/room is provided for patients and families to spend time together, and where private conversations can occur regarding care needs
16	Integrating a palliative care approach requires that patients with life limiting illness to receive compassionate end of life care
17	Integrating a palliative care approach requires care providers to update patients’ advance care preferences on a regular basis and post life threatening exacerbation
18	Clear systems and processes are in place for rapid end of life transfers to preferred place of care for those who are dying
19	All relevant clinical staff are aware of palliative community services when discharging a patient
20	The family are considered the unit of care, both informal and formal supports are available to the family and when this involves children age-appropriate information and support be available
21	Integrating a palliative care approach requires that formal mechanisms are in place in clinical departments/wards to facilitate shared decision making between doctors and nurses caring for patients/families with life limiting illness
22	Integrating a palliative care approach requires that formal mechanisms are in place in clinical departments/wards to ensure goals of care for patients with life limiting illness are clearly communicated between all staff caring for the patient and their family
23	The hospital has a multidisciplinary specialist palliative care service in place
24	A structured and standardised care pathway exists between relevant clinical staff and the specialist palliative care multi-disciplinary team, to support mutual decision making and organisation of care processes, including clarification of roles and responsibilities of care
25	The hospital has a multidisciplinary specialist palliative care consultation team in place, that is not only a ‘referral service’ but a service that includes consultations with relevant clinical staff
26	Specialist palliative care services have a clearly identified role in the continued training and support of other clinical staff
	Staff core indicators
27	Staff caring for patients with life limiting illness receive evidence-based training on palliative care principles, assessment and care management
28	Early career staff, and newly recruited staff caring for patients with life limiting illness receive evidence-based training on palliative care principles as part of induction training
29	Staff receive training on recognising and being able to assess the palliative care needs of patients
30	Staff receive training on a palliative care approach to patients’ pain and symptom control
31	Staff receive communication training regarding the needs of patients and their families with a life limiting illness
32	Staff receive training on compassionate end of life care

## Discussion

Consensus was achieved on 32 core indicators for the integration of a palliative care approach in hospitals. These reflect core activities at structural, clinical governance and individual practitioner level within this care environment. The need for multi-level engagement in the hospital setting in order to recognise and integrate palliative care has been identified in previous literature.^12,45–48^ The core indicators also reflect the need for a multidisciplinary approach to incorporate palliative care principles, with the adoption of clear care co-ordination, communication structures and shared decision making between care providers. These 32 indicators have broadened our understanding of what, at its core, access to and interaction between non-specialist and specialist palliative care providers should entail to support the integration of a palliative care approach in hospitals. This suggest that hospitals at a minimum should have a specialist palliative care team in place, which facilitates fluid and timely referrals and provides support, consultation, mentorship and training for staff. This reaffirms the perceived importance of specialist palliative care services for the effective integration of a palliative care approach in hospitals highlighted in previous literature.^49–51^ The importance of training on the recognition of those patients who may have palliative care needs, palliative care principles, assessment and care management for all care providers and in particular newly recruited staff and early career staff also emerged in the consensus process.

The integration of a palliative care approach, and the 32-core indicators within acute care provision in hospitals faces different challenges to that of specialist palliative care service integration. In models that focus on specialist palliative care integration^52^ the palliative care input is provided by those with a shared philosophy of palliative care working within the boundaries of specialist palliative care services and units, where the main palliative care role of healthcare providers working outside of specialist palliative care is considered referral to specialist palliative care. However, increasing the capacity of healthcare providers outside of specialist palliative care services to provide palliative care, as reflected in these core indicators, care providers within the acute biomedical ‘cure’ focused service provision in hospitals must assume a duality of roles by also integrating the fundamentally different view of ‘heal’ into their practice. The concern about whether it is feasible to incorporate a palliative care approach within hospitals has been raised by several authors.^12,53,54^ Adopting a whole system approach through the identification of structural, organisational and staff indicators may provide a means to recognise the need and support this integration in clinical practice.

The acute care environment however is a complex ecosystem.^55^ Even though individual hospital departments may serve separate functions in acute patient care, recognising that all healthcare providers working in hospitals have a role to play in the provision of a palliative care approach is essential.^10^ Exploration of a palliative care approach in previous studies within hospital departments such as the emergency department^56,57^ and intensive care^48,53^ support this, and highlight that while specific clinical priorities may occur between hospital departments, there is consistency in the fundamental palliative care philosophical approach to the patient. Consensus in this study was achieved on ‘what’ the core aspects of integrating a palliative care approach in hospitals requires through the identification of 32 core indicators. Further inquiry is required to establish ‘how’ they can be operationalised for use. This will likely require multi-level engagement involving rigorous examination of the core indicators within hospital structures, services and departments to ascertain how they may be integrated into a hospitals quality improvement system and quality standards reviews. Thus the 32 core indicators can be used as a basis to establish key outcome measures and quality indicators that could be used to evaluate the effectiveness and quality of an integrated palliative care approach in hospitals.

While 26 of the final 32 core indicators achieved consensus in all three stakeholder groups, indicating a high degree of consensus, 5 of the final core indicators did not achieve ⩾70% Agreement in the hospitalist stakeholder group and 1 did not achieve ⩾70% Agreement in the researcher stakeholder group. This suggests that differences exist between key stakeholders about the meaning and value of a palliative care approach in hospitals. The clinical application of these core indicators should be explored further from the perspective of how care is provided, and experienced by patients and families, recognising the variations and diversity that exists in palliative care provision in hospitals not only between countries, but also within-countries.^58^

### Strengths and limitations

The strengths of this study include the rigorous completion of two initial phases to identify a preliminary list of indicators of a palliative care approach.^8,9^ Also, of the 34 indicators that were presented for rating in round 1, 21 were retained following the final round indicating a high degree of stability between Delphi rounds. The inclusion of three diverse groups of international stakeholders’ views which included patients and families, and the overall retention rate of 74% represents further strengths of the findings. Several limitations are identified; participants were overwhelmingly from Ireland, and the findings are representative of the twelve countries who participated and may not be representative of other countries. Furthermore, the survey was only available online and in English, which may have limited the scope of participation.

### What this study adds?

These core indicators provide multi-level guidance to support the recognition and integration of a palliative care approach in hospitals. They provide a basis to assess, review and communicate the essential elements required to integrate a palliative care approach within hospitals. Contextual factors such as national palliative care policy, funding and organisation culture^58^ for example must be considered in relation to the clinical applicability and operationalisation of these core indicators in practice.

## Conclusions

The core indicators of a palliative care approach identified in this study reflect the fundamental palliative perspective of total pain and whole person care, highlighting the importance of a multidisciplinary palliative care approach in hospitals and the importance of the interface between non-specialist and specialist palliative care. One of the fundamental challenges for healthcare providers is recognition of the importance and value of integrating a palliative care approach within acute care. This study has established consensus on what the core aspects of a palliative care approach in acute care hospitals are. Further research is needed to understand how these core indicators of a palliative care approach can be operationalised for use in clinical practice.

## References

[1] Sleeman KE de Brito M Etkind S , et al The escalating global burden of serious health-related suffering: projections to 2060 by world regions, age groups, and health conditions. Lancet Global Health 2019; 7: e883–e892. 10.1016/S2214-109X(19)30172-X PMC6560023 31129125

[2] Sallnow L Smith R Ahmedzai SH , et al Report of the Lancet Commission on the Value of Death: bringing death back into life. Lancet 2022; 399: 837–884. 35114146 10.1016/S0140-6736(21)02314-X PMC8803389

[3] Gott M Robinson J. Are we getting it wrong? Perspectives on the future of palliative care in hospitals. Palliat Med. Epub ahead of 28 November 2018. DOI: 10.1177/0269216318809539. 30484379

[4] Broad JB Gott M Kim H , et al Where do people die? An international comparison of the percentage of deaths occurring in hospital and residential aged care settings in 45 populations, using published and available statistics. Int J Public Health 2013; 58: 257–267. 22892713 10.1007/s00038-012-0394-5

[5] Clark D Armstrong M Allan A , et al Imminence of death among hospital inpatients: Prevalent cohort study. Palliat Med 2014; 28: 474-479. 24637342 10.1177/0269216314526443 PMC4845030

[6] Cohen J Wilson D Thurston A , et al Access to palliative care services in hospital: a matter of being in the right hospital. Hospital charts study in a Canadian city. Palliat Med 2012; 26: 89–94. 21680750 10.1177/0269216311408992

[7] Higginson IJ Daveson BA Morrison RS , et al Social and clinical determinants of preferences and their achievement at the end of life: prospective cohort study of older adults receiving palliative care in three countries. BMC Geriatr 2017; 17: 271. 29169346 10.1186/s12877-017-0648-4 PMC5701500

[8] Nevin M Hynes G Smith V. Healthcare providers’ views and experiences of non-specialist palliative care in hospitals: a qualitative systematic review and thematic synthesis. Palliat Med 2020; 34: 605–618. 32020829 10.1177/0269216319899335

[9] Nevin M Smith V Hynes G. Non-specialist palliative care: a principle-based concept analysis. Palliat Med 2019; 33: 634–649. 31017529 10.1177/0269216319840963

[10] Ryan K Connolly M Charnley K , et al Palliative care competence framework. Dublin: Health Service Executive, 2014.

[11] Gardiner C Gott M Ingleton C , et al Extent of palliative care need in the acute hospital setting: a survey of two acute hospitals in the UK. Palliat Med 2013; 27: 76–83. 22619261 10.1177/0269216312447592

[bibr12-02692163241283540] Hynes G Kavanagh F Hogan C , et al Understanding the challenges of palliative care in everyday clinical practice: An example from a COPD action research project. Nurs Inq 2015; 22: 249–260. 25514830 10.1111/nin.12089

[bibr13-02692163241283540] Murray SA Boyd K. Using the ‘surprise question’ can identify people with advanced heart failure and COPD who would benefit from a palliative care approach. Palliat Med 2011; 25: 382. 21610113 10.1177/0269216311401949

[14] Safabakhsh N Lippe MP Govahi S , et al Unmet palliative care service needs: a patient-centred metric. BMJ Support Palliat Care. Epub ahead of print 22 February 2023. DOI: 10.1136/spcare-2023-004190. 36813534

[15] Seymour J Clark D. The Liverpool Care Pathway for the Dying Patient: a critical analysis of its rise, demise and legacy in England. Wellcome Open Res 2018; 3: 15. 29881785 10.12688/wellcomeopenres.13940.1 PMC5963294

[16] Bergenholtz H Jarlbaek L Hølge-Hazelton B. Generalist palliative care in hospital—Cultural and organisational interactions. Results of a mixed-methods study. Palliat Med 2016; 30: 558–566. 26643731 10.1177/0269216315619861

[bibr17-02692163241283540] Chan LS Macdonald ME Carnevale FA , et al ‘I’m only dealing with the acute issues’: How medical ward ‘busyness’ constrains care of the dying. Health 2018; 22: 451–468. 28552003 10.1177/1363459317708822

[18] Gott M Seymour J Ingleton C , et al ‘That’s part of everybody’s job’: the perspectives of health care staff in England and New Zealand on the meaning and remit of palliative care. Palliat Med 2012; 26: 232–241. 21677020 10.1177/0269216311408993

[19] Ingram C. A paradigm shift: healing, quality of life, and a professional choice. J Pain Sympt Manage 2014; 47: 198–201. 10.1016/j.jpainsymman.2013.09.015 24405630

[20] Hasselaar J Payne S. Moving the integration of palliative care from idea to practice. Palliat Med 2016; 30: 197–199. 26873983 10.1177/0269216315626039

[21] Gaertner J Siemens W Meerpohl JJ , et al Effect of specialist palliative care services on quality of life in adults with advanced incurable illness in hospital, hospice, or community settings: systematic review and meta-analysis. Br Med J 2017; 357: j2925. 10.1136/bmj.j2925 PMC5496011 28676557

[bibr22-02692163241283540] Groenvold M Petersen MA Damkier A , et al Randomised clinical trial of early specialist palliative care plus standard care versus standard care alone in patients with advanced cancer: The Danish Palliative Care Trial. Palliat Med 2017; 31: 814–824. 28494643 10.1177/0269216317705100

[23] Temel J Greer J Muzikansky A , et al Early palliative care for patients with metastatic non–small-cell lung cancer. N Engl J Med 2010; 363: 733–742. 20818875 10.1056/NEJMoa1000678

[24] Bergenholtz H Holge-Hazelton B Jarlbaek L. Organization and evaluation of generalist palliative care in a Danish hospital. BMC Palliat Care 2015; 14: 23. 25943367 10.1186/s12904-015-0022-2 PMC4431605

[bibr25-02692163241283540] Grubbs V Moss AH Cohen LM , et al A palliative approach to dialysis care: a patient-centered transition to the end of life. Clin J Am Soc Nephrol 2014; 9: 2203–2209. 25104274 10.2215/CJN.00650114 PMC4255391

[26] Shadd JD Burge F Stajduhar KI , et al Defining and measuring a palliative approach in primary care. Can Fam Physic 2013; 59: 1149–1150+1156–1157. PMC3828085 24235182

[27] The Centre for Clinical Governance Research in Health. Clinical indicators: a comprehensive review of the literature. Sydney: Centre for Clinical Governance Research in Health, University of New South Wales, 2009.

[28] De Roo ML Leemans K Claessen SJ , et al Quality indicators for palliative care: update of a systematic review. J Pain Sympt Manage 2013; 46: 556–572. 10.1016/j.jpainsymman.2012.09.013 23809769

[29] Twaddle ML Maxwell TL Cassel JB , et al Palliative care benchmarks from academic medical centers. Journal of Palliat Med 2007; 10: 86–98. 17298257 10.1089/jpm.2006.0048

[30] Mainz J. Developing evidence-based clinical indicators: a state of the art methods primer. Int J Qual Health Care 2003; 15: i5–i11. 14660518 10.1093/intqhc/mzg084

[31] Virdun C Button E Phillips JL , et al Perspectives of inpatients with palliative care needs, their families, clinicians and key stakeholders on measuring quality of hospital care via patient experience measures: a qualitative study. Palliat Med 2023; 37: 1498–1508. 37920917 10.1177/02692163231209845 PMC10657505

[32] Council of Europe. Recommendation Rec (2003) 24 of the Committee of Ministers to member states on the organisation of palliative care. Stockholm: Council of Europe, 2003.

[33] Frey R Gott M Banfield R. What indicators are measured by tools designed to address palliative care competence among ‘generalist’ palliative care providers? A critical literature review. Prog Palliat Care 2011; 19: 114–124.

[34] Junger S. Series: methodology in palliative medical research-The Delphi method: part 2. Palliat Med 2011; 12: 245–247.

[35] Hasson F Keeney S. Enhancing rigour in the Delphi technique research. Technol Forecast Soc Change 2011; 78: 1695–1704.

[36] Vernon W. The Delphi technique: a review. Int J Ther Rehabil 2009; 16: 69–76.

[37] Junger S Payne SA Brine J , et al Guidance on Conducting and REporting DElphi Studies (CREDES) in palliative care: recommendations based on a methodological systematic review. Palliat Med 2017; 31: 684–706. 28190381 10.1177/0269216317690685

[38] Boulkedid R Abdoul H Loustau M , et al Using and reporting the Delphi method for selecting healthcare quality indicators: a systematic review. PLoS ONE 2011; 6: e20476. 10.1371/journal.pone.0020476 PMC3111406 21694759

[39] Downar J Hawryluck L. What should we say when discussing “code status” and life support with a patient? A Delphi analysis. J Palliat Med 2010; 13: 185–195. 19929226 10.1089/jpm.2009.0269

[40] Mahler D Selecky P Harrod C. Management of dyspnea in patients with advanced lung or heart disease. Pol Arch Med Wewn 2010; 120: 160–166. 20502400

[41] Chesnay Md Roussel L Averill JB , et al Nursing research using data analysis. 1st ed. New York: Springer Publishing Company, 2014.

[42] Powell C. The Delphi technique: myths and realities. J Adv Nurs 2002; 41: 376–382. 10.1046/j.1365-2648.2003.02537.x 12581103

[43] Vázquez-Ramos R Leahy M Estrada Hernández N. The Delphi method in rehabilitation counseling research. Rehabil Counsel Bull 2016; 50: 111–118.

[44] Hsu C Sandford B. The Delphi technique: making sense of consensus. Pract Assess Res Evaluat 2007; 12: 1–8.

[45] Curry LA Brault MA Linnander EL , et al Influencing organisational culture to improve hospital performance in care of patients with acute myocardial infarction: a mixed-methods intervention study. BMJ Qual Saf 2018; 27: 207–217. 10.1136/bmjqs-2017-006989 PMC5867431 29101292

[bibr46-02692163241283540] Dalgaard KM Bergenholtz H Nielsen ME , et al Early integration of palliative care in hospitals: a systematic review on methods, barriers, and outcome. Palliat Support Care 2014; 12: 495–513. 24621947 10.1017/S1478951513001338

[bibr47-02692163241283540] Kamal AH Bowman B Ritchie CS. Identifying palliative care champions to promote high-quality care to those with serious illness. J Am Geriatr Soc 2019; 67: S461–S467. 10.1111/jgs.15799 31074852

[48] Mosenthal AC Murphy PA Barker LK , et al Changing the culture around end-of-life care in the trauma intensive care unit. J Trauma 2008; 64: 1587–1593. 18545128 10.1097/TA.0b013e318174f112

[49] Firn J Preston N Walshe C. What are the views of hospital-based generalist palliative care professionals on what facilitates or hinders collaboration with in-patient specialist palliative care teams? A systematically constructed narrative synthesis. Palliat Med 2016; 30: 240–256. 26873984 10.1177/0269216315615483

[bibr50-02692163241283540] Health Service Executive. Palliative care operational plan. Dublin: Health Service Executive, 2015.

[51] Robinson J Gott M Gardiner C , et al The ‘problematisation’ of palliative care in hospital: an exploratory review of international palliative care policy in five countries. BMC Palliat Care 2016; 15: 64. 27456495 10.1186/s12904-016-0137-0 PMC4960865

[52] Siouta N Van Beek K van der Eerden ME , et al Integrated palliative care in Europe: a qualitative systematic literature review of empirically-tested models in cancer and chronic disease. BMC Palliat Care 2016; 15: 56. 27391378 10.1186/s12904-016-0130-7 PMC4939056

[53] Gélinas C Fillion L Robitaille M-A , et al Stressors experienced by nurses providing end-of-life palliative care in the intensive care unit. Can J Nurs Res 2012; 44: 18–39. 22679843

[54] Glogowska M Simmonds R McLachlan S , et al “Sometimes we can’t fix things”: a qualitative study of health care professionals’ perceptions of end of life care for patients with heart failure. BMC Palliat Care 2016; 15: 3. 26762266 10.1186/s12904-016-0074-y PMC4712523

[55] Nadalin Penno L Davies B Graham ID , et al Identifying relevant concepts and factors for the sustainability of evidence-based practices within acute care contexts: a systematic review and theory analysis of selected sustainability frameworks. Implement Sci 2019; 14: 108. 31856861 10.1186/s13012-019-0952-9 PMC6923954

[56] Cooper E Hutchinson A Sheikh Z , et al Palliative care in the emergency department: a systematic literature qualitative review and thematic synthesis. Palliat Med 2018; 32: 1443–1454. 30028242 10.1177/0269216318783920

[57] Smith AK Schonberg MA Fisher J , et al Emergency department experiences of acutely symptomatic patients with terminal illness and their family caregivers. J Pain Sympt Manage 2010; 39: 972–981. 10.1016/j.jpainsymman.2009.10.004 PMC2884054 20434873

[58] Zaman S Inbadas H Whitelaw A , et al Common or multiple futures for end of life care around the world? Ideas from the ‘waiting room of history’. Soc Sci Med 2017; 172: 72–79. 27894008 10.1016/j.socscimed.2016.11.012 PMC5224187

